# Green Synthesis of Copper Oxide Nanoparticles Using *Citrus sinensis* Leaves: Effects of Experimental Parameters, Antimicrobial Evaluation and Development of Chitosan Composites

**DOI:** 10.3390/nano16060369

**Published:** 2026-03-18

**Authors:** Jordana Bortoluz, Axel J. P. Jacquot, Lucas C. Colissi, Paula Sartori, Lílian V. R. Beltrami, Régis Guégan, Giovanna Machado, Mariana Roesch-Ely, Janaina S. Crespo, Marcelo Giovanela

**Affiliations:** 1 Programa de Pós-Graduação em Engenharia e Ciência dos Materiais, Área do Conhecimento de Ciências Exatas e Engenharias, Universidade de Caxias do Sul, Rua Francisco Getúlio Vargas, 1130, Caxias do Sul 95070-560, RS, Brazil; jacquot.axel.bj@gmail.com (A.J.P.J.); lccolissi@ucs.br (L.C.C.); jscrespo@ucs.br (J.S.C.); 2 Unidade de Pesquisa e Desenvolvimento UCSGRAPHENE^®^, Universidade de Caxias do Sul, Rua Francisco Getúlio Vargas, 1130, Caxias do Sul 95070-560, RS, Brazil; psartori1@ucs.br; 3 École Européenne d’Ingénieurs en Génie des Matériaux, Université de Lorraine, 6 rue Bastien-Lepage, BP 10630, F-54010 Nancy CEDEX, France; 4 Instituto de Biotecnologia, Área do Conhecimento de Ciências da Vida, Universidade de Caxias do Sul, Rua Francisco Getúlio Vargas, 1130, Caxias do Sul 95070-560, RS, Brazil; mrely@ucs.br; 5 Programa de Pós-Graduação em Engenharia de Processos e Tecnologias, Área do Conhecimento de Ciências Exatas e Engenharias, Universidade de Caxias do Sul, Rua Francisco Getúlio Vargas, 1130, Caxias do Sul 95070-560, RS, Brazil; lvrossa@ucs.br; 6 Interfaces, Confinement, Matériaux et Nanostructures (ICMN), UMR 7374, CNRS-Université d’Orléans, 3 Avenue de la Recherche Scientifique, 45071 Orléans CEDEX 2, France; regis.guegan@univ-orleans.fr; 7 Laboratório de Materiais Nanoestruturados (LMNANO), Centro de Tecnologias Estratégicas do Nordeste (CETENE), Av. Prof. Luís Freire, 1, Cidade Universitária, Recife 50740-545, PE, Brazil; giovanna.machado@cetene.gov.br

**Keywords:** *Citrus sinensis*, green synthesis, CuONPs, physicochemical characterization, antimicrobial properties, chitosan, composites

## Abstract

Copper oxide nanoparticles (CuONPs) have received considerable attention because of their wide range of applications, particularly in the development of antimicrobial materials for medical, environmental, and industrial purposes. However, conventional synthesis routes often involve the use of toxic chemicals and environmentally harmful conditions. To overcome these limitations, green synthesis strategies have been developed as sustainable alternatives through the use of natural reducing and stabilizing agents. In this study, *Citrus sinensis* leaf extract, which exhibits high antioxidant capacity, was investigated for green synthesis of CuONPs, followed by their subsequent incorporation into a chitosan polymeric matrix. The optimal synthesis conditions were achieved at a pH of 7.0 using copper(II) acetate monohydrate (Cu(CH_3_COO)_2_·H_2_O) at a concentration of 10.0 g L^−1^ and a calcination temperature of 300 °C. The resulting CuONPs exhibited a heterogeneous morphology, with average particle sizes ranging from 20 to 30 nm, and demonstrated satisfactory antimicrobial activity against *Escherichia coli* and *Staphylococcus aureus*. The incorporation of these NPs into chitosan yielded composite materials with enhanced antimicrobial performance, highlighting the added value of polymer–NP hybrid systems. Although these composite materials were not evaluated under realistic operational conditions, the optimized green protocol provides a robust methodological basis for future studies targeting water disinfection and other environmentally relevant technologies.

## 1. Introduction

Copper oxide nanoparticles (CuONPs) are widely recognized for their stability, long shelf life, and simple production process [1,2]. Compared with other NPs, CuONPs exhibit lower toxicity to living cells, rendering them particularly appealing for biomedical and environmental applications. Consequently, these NPs have been extensively investigated for their antidiabetic, antioxidant, anticancer, antifungal, and antimicrobial properties [3,4].

Beyond the biomedical field, the semiconductor nature of CuONPs enables their use in various technological domains, including the production of electrically conductive materials, solar energy cells, gas sensors, and fungal infestation detection systems [2,5]. Additionally, they can act as efficient catalysts in high-temperature and high-pressure chemical reactions [2,6]. From an environmental perspective, CuONPs have also been investigated as nanoadsorbents for industrial wastewater remediation purposes [3,4,5]. Furthermore, their potential to mitigate bacterial resistance highlights their relevance in addressing current challenges in antimicrobial research [7].

Although CuONPs can be synthesized through various techniques, conventional chemical and physical methods may present important limitations. Chemical routes often rely on strong reducing agents, stabilizers, or organic solvents that can increase the environmental footprint of the synthesis process [8]. In many wet-chemical approaches, reagents such as hydrazine, sodium borohydride, or other synthetic reducing agents are employed, which require careful handling and may generate chemical residues that require further treatment [9,10,11,12,13]. In contrast, several physical methods typically demand high energy input, specialized equipment, or elevated temperatures, which may limit their scalability and sustainability [14].

In this context, plant-mediated synthesis routes have therefore emerged as a promising alternative, aiming to reduce the use of auxiliary reagents by employing natural extracts that can simultaneously act as reducing and stabilizing agents, while also controlling particle size through complexation mechanisms [15,16]. It is important to emphasize that these approaches do not completely eliminate chemically derived reagents, since metal precursor salts are still required as a source of metal ions. Furthermore, pH adjustments may be necessary using chemical agents such as sodium hydroxide, as variations in pH can modify the chemical species present in plant extracts, thereby facilitating their interaction with metal ions from the precursor salt and promoting NP formation. Nevertheless, plant-mediated synthesis can simplify reaction systems and enable NP production under relatively mild experimental conditions [17,18].

Due to their accessibility, safety, and sustainability, plants are among the most widely adopted materials in green synthesis. Unlike bacteria and fungi, they pose minimal handling risks and offer economic and environmental advantages [19]. Various plant-derived materials have been successfully employed in the green synthesis of CuONPs, including *Simarouba glauca* leaves [5], *Punica granatum* bark [20], *Hyphaene thebaica* fruits [21], *Berberis lycium* roots [22], and raw garlic (*Allium sativum*) [3]. Although the exact mechanism of plant-mediated synthesis remains under investigation, plants are known to contain a diverse array of secondary metabolites, including flavonoids, sugars, proteins, tannins, phenols, and terpenoids [19,23,24,25]. These compounds act as effective reducing agents, promoting the reduction of copper ions from precursor salts, which are frequently applied for this purpose. Following synthesis, the resulting copper complexes are thermally decomposed under high temperatures to yield CuONPs [15,26].

Within this context, orange (*Citrus sinensis*) leaves have emerged as a promising natural source for green synthesis of CuONPs because of their high content of flavonoids and other phenolic compounds, which possess notable antioxidant properties [27,28]. Furthermore, orange trees are widely cultivated worldwide, particularly in tropical and subtropical regions, and play a crucial role in driving the global economy through the production of fruits, juice, and essential oils [27,29]. The valorization of agricultural residues, such as citrus leaves, therefore represents an attractive strategy for developing sustainable routes for nanomaterial synthesis.

While numerous studies have reported the green synthesis of metal oxide NPs using plant extracts, many of these works focus primarily on demonstrating NP formation and basic biological activity [30]. In contrast, systematic investigations addressing the influence of synthesis parameters, the mechanistic aspects of NP formation, and the integration of these NPs into functional materials remain relatively limited. A deeper understanding of these aspects is essential for improving the reproducibility and functional performance of plant-mediated NP synthesis routes.

Considering these aspects and the limited number of systematic studies on the green synthesis of CuONPs using *C. sinensis* leaf extract, this work aimed to (i) investigate the synthesis of these NPs and the influence of key parameters on their antimicrobial activity, and (ii) evaluate their immobilization in a chitosan polymeric matrix to develop materials with antimicrobial properties. In addition to assessing synthesis conditions, the present study also investigates the possible formation mechanism of CuONPs using electrochemical analysis. These findings may contribute to advancing sustainable NP synthesis methodologies and expanding their potential applications in strategically relevant fields.

## 2. Materials and Methods

### 2.1. Materials

*C. sinensis* leaves used for extract preparation were collected in the municipality of Caxias do Sul (geographic coordinates: 29.191117° S, 51.186857° W), located in Rio Grande do Sul state, southern Brazil. The leaf extract was characterized using Folin–Ciocalteu reagent 2N (Dinâmica Química Contemporânea Ltd., Indaiatuba, Brazil), calcium carbonate P.A. (Dinâmica Química Contemporânea Ltd., Indaiatuba, Brazil), tris(hydroxymethyl)aminomethane hydrochloride (Tris-HCl) (≥99.0%, Sigma-Aldrich, São Paulo, Brazil), 1,1-diphenyl-2-picrylhydrazyl (DPPH, Sigma-Aldrich, São Paulo, Brazil), and ethanol 99% (Dinâmica Química Contemporânea Ltd., Indaiatuba, Brazil). Green synthesis was conducted using copper(II) nitrate trihydrate (Cu(NO_3_)_2_·3H_2_O, ≥99.0%, Sigma-Aldrich, São Paulo, Brazil) or copper(II) acetate monohydrate (Cu(CH_3_COO)_2_·H_2_O, ≥98.0%, Sigma-Aldrich, São Paulo, Brazil) and deionized water obtained from a Millipore Direct-Q 3 ultraviolet (UV) system (Merck Group, Darmstadt, Germany). Chitosan (as coarse flakes and powder with high molecular weights; Sigma-Aldrich, São Paulo, Brazil), glacial acetic acid (99.7% *v*/*v*; Dinâmica Química Contemporânea Ltd., IndaiatubaP, Brazil), and glutaraldehyde (50% *v*/*v*; Dinâmica Química Contemporânea Ltd., Indaiatuba, Brazil) were employed in the preparation of chitosan–CuONP composites. The antimicrobial activity of the prepared CuONPs was evaluated against *Escherichia coli* (*E. coli*, Gram-negative, ATCC, 25922) and *Staphylococcus aureus* (*S. aureus*, Gram-positive, ATCC, 25923). Mueller–Hinton agar (K25-1058, Kasvi, Pinhais, Brazil) and Mueller–Hinton broth (K25-1214, Kasvi, Pinhais, Brazil) were chosen as growth media for both bacteria.

### 2.2. Citrus sinensis Leaf Extract Preparation

*C. sinensis* leaves were manually collected and thoroughly washed with tap water to remove surface contaminants. A portion of the fresh leaves was immediately ground and used in their natural state, while the remaining portion was subjected to a drying process. Drying was performed in an oven at 45 °C for 8 h. After drying, the leaves were ground into a fine powder using a blender. The resulting leaf powder was stored at room temperature in a desiccator protected from light until further use.

Extracts were prepared via the maceration technique [31]. Predetermined amounts of fresh or dried leaves were mixed with the selected solvent in a beaker and maintained under magnetic stirring for a defined extraction period. At the end of this process, the mixtures were filtered to obtain extracts. The evaluated extraction conditions, along with the corresponding experimental parameters for each test, are summarized in Table 1. All extraction experiments were performed in triplicate. The Folin–Ciocalteu method was used to quantify the total phenolic compounds present in the plant extracts obtained under different extraction conditions, allowing the identification of the optimal conditions for subsequent experiments. This method is based on the ability of phenolic compounds present in the plant extract to reduce molybdenum ions found in the Folin–Ciocalteu reagent, resulting in the formation of coordination complexes that cause a color change in the solution from yellow to blue. The intensity of the blue coloration formed is proportional to the concentration of phenolic compounds present in the extract and is therefore used for their spectrophotometric quantification [32].

#### 2.2.1. Total Phenolic Content in *Citrus sinensis* Leaf Extract

The total phenolic content was determined according to a procedure adapted from the literature [33]. Briefly, 150 µL of extract was mixed with 750 µL of Folin–Ciocalteu reagent (10% *v*/*v*) and 600 µL of sodium carbonate (7.5% *m*/*v*), followed by incubation at 55 °C for 5 min. Quantification was performed using a UV-2600i spectrophotometer (Shimadzu, Kyoto, Japan) on the basis of an external calibration curve constructed with six known concentrations of gallic acid (GA) within the concentration range of 1.5–100.0 μg mL^−1^, prepared from a 100 µg mL^−1^ stock solution in an ethanol-deionized water mixture (1:1 *v*/*v*). The absorbance was measured at a wavelength of 760 nm, and the results are expressed as micrograms of GA equivalents per milliliter of extract (µg GAE mL^−1^) and milligrams of GA equivalents per gram of leaves (mg GAE g^−1^).

#### 2.2.2. Antioxidant Activity of *Citrus sinensis* Leaf Extract

The antioxidant activity of the extract obtained under the optimized conditions was evaluated using a DPPH radical scavenging assay. In this method, the antioxidant compounds present in the extract reduce the DPPH radical, resulting in a color change from violet to yellow.

The DPPH assay was performed according to the procedure described by Yamaguchi et al. [34], with minor adaptations. Briefly, 100 μL of the pure extract was mixed with 400 μL of a 0.1 mol L^−1^ Tris-HCl solution (pH 7.0) and 500 μL of a 0.5 mmol L^−1^ DPPH solution in test tubes. The control sample was prepared by replacing the extract with the corresponding solvent. After homogenization, the samples were stored in the dark for 20 min. The absorbance was measured at a wavelength of 517 nm using a UV-2600i spectrophotometer. The inhibition percentage was calculated according to Equation (1):

(1) $$\%inhibition=\frac{A_{C}-A_{S}}{A_{C}}\times 100\%$$

where *A_C_* and *A_S_* denote the absorbance values of the control and the sample containing the extract, respectively.

### 2.3. CuONP Green Synthesis

Similar to the extract preparation process, several experimental parameters were systematically evaluated and optimized for green synthesis of CuONPs. In a typical procedure, 175 mL of an aqueous solution of either Cu(NO_3_)_2_·3H_2_O or Cu(CH_3_COO)_2_·H_2_O at predefined concentrations was mixed with 25 mL of pure *C. sinensis* leaf extract and heated to 70 °C. During synthesis, the reaction temperature was controlled between 70 and 80 °C using a water bath maintained. An ultrathermostatic bath set to a temperature of 15 °C was employed to circulate the coolant through the condensers attached to the flask, thereby preventing solvent evaporation.

In plant-mediated NP synthesis routes, relatively large volumes of extract are commonly employed because plant metabolites act simultaneously as reducing, complexing, and stabilizing agents. Unlike conventional chemical synthesis routes that rely on reducing agents with defined stoichiometry, plant extracts contain complex mixtures of phenolics, flavonoids, sugars, and other secondary metabolites whose effective concentrations are difficult to quantify precisely [23,24,25]. For this reason, an excess of extract is typically used to ensure sufficient availability of reducing and stabilizing species during NP formation.

At the end of the reaction, the synthesized NPs were separated via centrifugation at 5000 rpm for 10 min. The supernatant was discarded, and the solid product was washed with 200 mL of deionized water to remove unreacted species and other impurities. The recovered material was subsequently dried in an oven at 100 °C for 1 h and preground with a mortar and pestle. Finally, the dried powder was calcined under controlled conditions and ground again to obtain the final CuONPs.

Green synthesis of CuONPs from *C. sinensis* leaf extract was investigated through fourteen distinct experimental methods, with various key parameters such as the type and concentration of copper precursor salt, solution pH, and calcination temperature. After each synthesis, X-ray diffraction (XRD) analysis was performed to confirm the formation and crystalline profile of the obtained CuONPs. In addition, bacterial growth inhibition assays were conducted to assess the suitability of the synthesized NPs for antimicrobial applications. The experimental conditions evaluated under each synthesis route are summarized in Table 2.

### 2.4. Investigation of the Formation Mechanism of CuONPs

To better understand the electrochemical behavior of copper species during the synthesis process, different reaction media were evaluated, including the plant extract, the pure precursor salt solution, the reaction mixture obtained from the synthesis without pH adjustment, and the reaction mixture obtained under the optimal pH conditions.

The electrochemical reactions associated with copper species are described by Equations (2)–(5), corresponding to the Cu(II)/Cu(I)/Cu(0) redox couples. These reactions indicate that any dissolved copper species present in the solution can be identified through their characteristic electrochemical responses.

(2) $${Cu}^{+}+e^{-}\to {Cu}^{0}\;-0.427\;V$$

(3) $${Cu}^{0}\to {Cu}^{+}+e^{-}\;-0.101\;V$$

(4) $${Cu}^{2+}+e^{-}\to {Cu}^{+}\;+0.127\;V$$

(5) $${Cu}^{+}\to {Cu}^{2+}+e^{-}\;+0.222\;V$$

The formation mechanism of CuONPs mediated by *C. sinensis* leaf extract was investigated by cyclic voltammetry using a conventional three-electrode electrochemical cell at room temperature. Measurements were performed with a potentiostat (IviumStat A11701, Ivium Technologies, Eindhoven, The Netherlands) over a potential window ranging from −0.9 to +0.9 V at a scan rate of 50.0 mV s^−1^ and a step potential of 5.0 mV. Cyclic voltammograms were recorded via the use of a glassy carbon working electrode, a platinum wire counter electrode, and a calomel reference electrode. Prior to each analysis, the working electrode surface was polished with an alumina slurry and subsequently sonicated in acetone for 5 min to ensure reproducible electrochemical responses. A 1.0 mol L^−1^ KCl aqueous solution was employed as the supporting electrolyte in all the experiments.

### 2.5. Characterization of CuONPs

#### 2.5.1. X-Ray Diffraction (XRD)

XRD diffractograms of the CuONPs were recorded using an XRD-6000 powder diffractometer (Shimadzu, Kyoto, Japan) equipped with a Cu *K_α_*_1_ radiation source (λ = 1.5406 Å). Data were collected over a 2θ range from 20° to 80°, with a counting time of 5 s per step. Diffractogram analysis and phase identification were performed using QualX software.

#### 2.5.2. Field Emission Scanning Electron Microscopy (FESEM) Coupled with Energy Dispersive Spectroscopy (EDS)

The morphology of the synthesized CuONPs was initially evaluated via FESEM. Prior to analysis, the NPs were sputter-coated with a thin gold layer for approximately 10 s. Micrographs were acquired using a MIRA3 electron microscope (TESCAN, Brno, Czech Republic) operated at an accelerating voltage of 20 kV. Elemental composition analysis was conducted via an EDS device coupled to the FESEM system using a silicon drift detector.

#### 2.5.3. Transmission Electron Microscopy (TEM)

In TEM analysis, the CuONPs were ultrasonically dispersed in isopropanol for 30 min and subsequently deposited onto copper grids. TEM images were obtained using an FEI Morgagni 268D transmission electron microscope (Thermo Fisher Scientific, Waltham, MA, USA) operating at an accelerating voltage of 80 kV. The average particle size was determined using ImageJ 1.53 software by measuring approximately 150 individual particles [35].

### 2.6. Antimicrobial Tests

The antimicrobial activity of CuONPs was evaluated in both solid and liquid media following the Clinical and Laboratory Standards Institute (CLSI) M2-A8 (disc diffusion method) [36] and CLSI M7-A6 (broth dilution testing) [37] standards, respectively. These assays were performed to assess the bactericidal and/or bacteriostatic effects of the synthesized NPs against *E. coli* and *S. aureus*. Prior to testing, all CuONPs were sterilized under UV irradiation for 8 h.

The bacteriostatic activity in the solid medium was evaluated using Muller–Hinton agar plates. Bacterial strains were inoculated at a concentration of 1.0 × 10^6^ colony-forming units per milliliter (CFU mL^−1^), and 10.0 mg of CuONPs was added to the surface of each plate. The plates were then incubated at 37 °C for 24 h, after which the presence or absence of inhibition zones surrounding the NPs was visually assessed and documented photographically. For the liquid medium assays, bacterial strains were prepared at a concentration of 1.0 × 10^4^ CFU mL^−1^ and inoculated into 12-well plates containing Mueller–Hinton broth. As in the solid medium tests, 10.0 mg of CuONPs was added to each well. The plates were incubated at 37 °C for 24 h. The following day, aliquots from each well were collected with sterile swabs and streaked onto Mueller–Hinton agar plates, which were subsequently incubated at 37 °C for an additional 24 h. Bacterial growth was subsequently evaluated and documented photographically.

### 2.7. Preparation of Chitosan–CuONP Composites

CuONPs were incorporated into a chitosan polymeric matrix to produce pellet-shaped composite materials. First, 1.5 g of chitosan was dissolved in 100 mL of a 0.75 mol L^−1^ glacial acetic acid aqueous solution. The mixture was magnetically stirred for 15 min and then left to stand for 24 h to ensure complete polymer dissolution and removal of air bubbles formed in the process. Subsequently, 7.0 g of the resulting chitosan solution was mixed with 0.1 g of CuONPs. The composite pellets were prepared via dropwise injection of the mixture into 100 mL of a 1.0 mol L^−1^ NaOH aqueous solution containing 60 µL of glutaraldehyde, which served as the crosslinking agent. Chitosan pellets without CuONPs were also prepared under identical conditions and employed as control samples to assess the contribution of NPs to the antimicrobial performance of the composites. As described in the following sections, only CuONPs synthesized under the optimal conditions were selected for further characterization and subsequent incorporation into the chitosan polymeric matrix.

## 3. Results and Discussion

### 3.1. Preparation and Characterization of Citrus sinensis Leaf Extract

#### 3.1.1. Total Phenolics

Preliminary tests were conducted to identify the optimal conditions for extracting bioactive compounds from *C. sinensis* leaves, which play a key role in determining the effectiveness of green synthesis of CuONPs. As these leaves contain abundant phenolic compounds, the extraction efficiency was evaluated via the Folin–Ciocalteu method. According to Azmir et al. [31], factors such as plant pretreatment, solvent type, temperature, and extraction time notably influence the extraction of phenolic compounds. Because both fresh and dried plant materials have been used in plant-mediated nanoparticle synthesis reported in the literature, the influence of leaf pretreatment was also experimentally evaluated in this study. For these reasons, all these parameters were systematically investigated in this study.

The results of Test 1, which aimed to examine the influences of leaf pretreatment and solvent type, are listed in Table 3. Notably, the phenolic contents in the extracts prepared using dried and ground leaves were greater than those in the extracts prepared using fresh leaves, regardless of the extraction solution. Although drying may lead to partial degradation of select compounds, the combined drying and grinding process facilitates particle size reduction and increases the surface area, thus enhancing solvent–solid interactions and the extraction efficiency [31]. In addition, drying removes a significant fraction of water naturally present in fresh leaves. Therefore, equal masses of fresh and dried samples do not contain equivalent amounts of phenolic compounds.

The use of drying and grinding as pretreatment steps is consistent with previous studies involving citrus leaves. For example, Singh et al. [38] prepared *Origanum majorana* and *C. sinensis* extracts for the synthesis of silver nanoparticles (AgNPs) using sun-dried plant material, whereas Ekwenye and Edeha [39] evaluated the antimicrobial activity of *C. sinensis* leaf extracts obtained from freshly harvested leaves that were washed with tap water and dried in an oven at 65 °C for 36 h. Similarly, Lagha-Benamrouche and Madani [28] investigated the phenolic composition and antioxidant activity of *C. sinensis L.* and *C. aurantium*, thereby employing leaves that were cut and dried in an oven at 40 °C for one week prior to analysis.

Solvent selection represents another critical parameter in the extraction process. Although aqueous mixtures of organic solvents are commonly reported to be effective for the extraction of phenolic compounds [40], the principles of green chemistry encourage the use of solvents that are safe, environmentally sustainable, and economically viable. Based on these considerations, only two extraction systems were evaluated in this study: an ethanol/deionized water mixture (1:1, *v*/*v*) and deionized water heated to 70 °C.

As indicated in Table 3, extraction using deionized water at 70 °C resulted in a slightly higher average phenolic content (400.90 µg GAE mL^−1^ of extract) than that obtained with the ethanol–water mixture (386.49 µg GAE mL^−1^). This result suggests that most phenolic compounds in *C. sinensis* leaves, including anthocyanins, tannins, saponins, and terpenoids, exhibit greater affinity for water than for ethanol [31]. Although the phenolic content may vary with factors such as the harvest period and plant maturity, the use of water is particularly advantageous because of its alignment with green chemistry principles and its environmental and economic benefits. Similar behavior was reported by Adnan et al. [41], who revealed that compared with methanol, distilled water heated to 50 °C extracted greater amounts of phenolic compounds from the leaves of *C. paradisi*, *C. sinensis*, and *C. jambhiri* at different concentrations (50–100% *v*/*v*). With respect to *C. sinensis*, the reported phenolic contents ranged from 608 to 1379.3 mg GAE g^−1^, depending on the solvent composition.

After the optimal leaf pretreatment and solvent type were established, the effect of the extraction temperature was investigated, as detailed in Test 2 (Table 3). Deionized water with a temperature of 25, 45, or 70 °C was used. Notably, increasing the temperature enhanced phenolic compound extraction. Elevated temperatures promote tissue softening and cell membrane disruption, thus facilitating solvent penetration and phenolic release [42]. In addition, higher temperatures increase molecular motion, reduce the solvent viscosity, and lower surface tension, thereby enhancing mass transfer and the extraction efficiency [43]. Despite the positive effect of temperature, excessive heating may lead to solvent loss and degradation of thermolabile phenolic compounds. Antony and Farid [44] reported that maximum phenolic yields in conventional extraction processes typically occur between 60 and 80 °C, whereas Akowuah et al. [45] reported the degradation of sensitive phenolics at elevated temperatures. On the basis of these considerations, an extraction temperature of 70 °C was selected for the subsequent experiments.

The last parameter evaluated was the contact time between *C. sinensis* leaf powder and the solvent, hereafter referred to as Test 3 (Table 3). In the initial experiments, the extraction time was set to 30 min on the basis of previous studies revealing effective phenolic extraction within short contact times [3,36]. To optimize the process, shorter extraction times (5 and 10 min) were investigated. Shorter extraction times resulted in an average phenolic concentration of approximately 325 µg GAE mL^−1^, whereas extending the extraction process to 30 min increased this value to approximately 400.90 µg GAE mL^−1^. Consequently, a 30-min extraction time was maintained to maximize the phenolic yield, given the central role of these compounds in driving the green synthesis of CuONPs.

Finally, the results of the Folin–Ciocalteu method revealed that under the optimized extraction experimental conditions, a total phenolic content of approximately 400.90 µg GAE mL^−1^ in the extract could be achieved, corresponding to 26.73 mg GAE g^−1^ in *C. sinensis* leaves. Although this value is lower than that reported by Adnan et al. [41] (1379.3 mg GAE g^−1^ in *C. sinensis* leaves), it suitably agrees with the phenolic content reported by Tawaha et al. [46] (23.4 mg GAE g^−1^ in *C. sinensis* leaves).

Although phenolic compounds were used in this study as an indicator of the reducing potential of the extract, it should be noted that plant extracts contain a complex mixture of secondary metabolites, including flavonoids, sugars, terpenoids, proteins, and organic acids such as ascorbic acid [25]. Many of these compounds may also participate in the reduction and stabilization of metal ions during NP formation. In addition to their reducing ability, phenolic compounds and flavonoids can interact with NP surfaces through hydroxyl and carbonyl functional groups, acting as capping agents that influence particle growth, aggregation behavior, and final particle size distribution [47,48].

#### 3.1.2. Antioxidant Activity

Another relevant characteristic of the extract for green synthesis applications is its antioxidant capacity, as this property is directly associated with the ability of plant metabolites to serve as reducing agents during NP formation. To evaluate this behavior, a DPPH radical scavenging assay was employed. The extracts used in this analysis were prepared under the optimized conditions previously identified via the Folin–Ciocalteu method.

On the basis of Equation (1), the *C. sinensis* leaf extract exhibited a DPPH radical scavenging activity of approximately 80%, indicating a high content of antioxidant compounds. This result is consistent with the findings reported by Khettal et al. [49], who investigated the antioxidant activity of several leaf extracts and reported DPPH scavenging values ranging from 48 to 84%. In that study, different extraction methods and solvents were evaluated, and the results indicated that the aqueous extracts of *C. aurantifolia*, *C. hamlin*, and *C. limon* generally exhibited higher antioxidant activity than their methanolic counterparts did, whereas no significant differences were observed between the aqueous and methanolic extracts of *C. naval*, *C. grandi*, and *C. clementina*. These observations support the high antioxidant capacity of *C. sinensis* leaf extract and its suitability for use as a reducing agent in green synthesis of CuONPs.

### 3.2. Green Synthesis of CuONPs Based on Citrus sinensis Leaf Extract and Their Antimicrobial Properties

The main experimental parameters governing the green synthesis of CuONPs, including the pH, copper precursor type and concentration, were systematically investigated, thereby maintaining the *C. sinensis* leaf extract concentration constant at 0.1 g mL^−1^. The results demonstrated that synthesis performed at a pH of 7.0 led to the formation of CuO phases, with the final composition highly dependent on the nature of the copper precursor salt. In addition, when Cu(CH_3_COO)_2_·H_2_O was employed as the precursor, the formation of a CuO-dominant phase was observed. Conversely, synthesis using Cu(NO_3_)_2_·3H_2_O under the same conditions resulted in the simultaneous formation of CuO and Cu_2_O phases. In the latter system, the calcination temperature played a decisive role in determining the relative proportion of Cu_2_O, with higher temperatures promoting its gradual conversion to CuO. A comprehensive summary of all the experimental conditions is provided in the Table 2 and Figure S1 (Supplementary Material).

**Figure 1:**
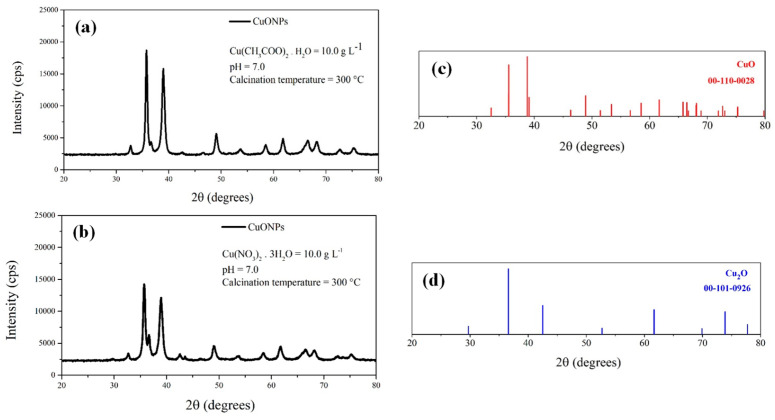
XRD patterns of CuONPs synthesized at a pH of 7.0 and calcined at 300 °C using different copper precursors at a concentration of 10.0 g L^−1^: (**a**) Cu(CH_3_COO)_2_·H_2_O; (**b**) Cu(NO_3_)_2_·3H_2_O. Reference diffraction patterns of (**c**) CuO and (**d**) Cu_2_O are shown for comparison.

Figure 1 shows the XRD patterns of the CuONPs synthesized under different conditions, along with the reference diffraction patterns used for phase identification. The figure is divided into four parts: (a) XRD patterns of CuONPs synthesized using Cu(CH_3_COO)_2_·H_2_O as precursor; (b) XRD patterns of CuONPs synthesized using Cu(NO_3_)_2_·3H_2_O as precursor; (c) the reference diffraction pattern corresponding to CuO peaks obtained from the software database; and (d) the reference diffraction pattern corresponding to Cu_2_O peaks. The diffraction peaks observed in the synthesized samples were compared with the reference patterns to identify the crystalline phases present.

Although the samples synthesized from Cu(NO_3_)_2_·3H_2_O still exhibited minor Bragg reflections associated with the Cu_2_O phase, their intensity was significantly lower than that observed for samples calcined at 200 °C (Figure S2, Supplementary Material), indicating a higher degree of conversion to CuO at the higher calcination temperature. These results demonstrate that calcination at 300 °C effectively promotes the formation of CuO-dominant NPs. Based on these findings, the optimal experimental parameters for CuONP synthesis corresponded to pH 7.0, a copper precursor concentration of 10.0 g L^−1^, and a calcination temperature of 300 °C. It should be noted that the presence of minor amounts of Cu_2_O does not necessarily compromise the antimicrobial performance of copper-based nanomaterials. Both CuO and Cu_2_O NPs have been widely reported to exhibit antimicrobial activity, which is generally attributed to mechanisms such as copper ion release, generation of reactive oxygen species (ROS), and interactions with bacterial cell membranes [50]. In some cases, the coexistence of Cu(I) and Cu(II) species has even been suggested to enhance antimicrobial effects due to redox cycling processes that promote oxidative stress in microbial cells. Therefore, the presence of small amounts of Cu_2_O may contribute to the overall biological activity of the material rather than representing a detrimental factor [51].

**Figure 2:**
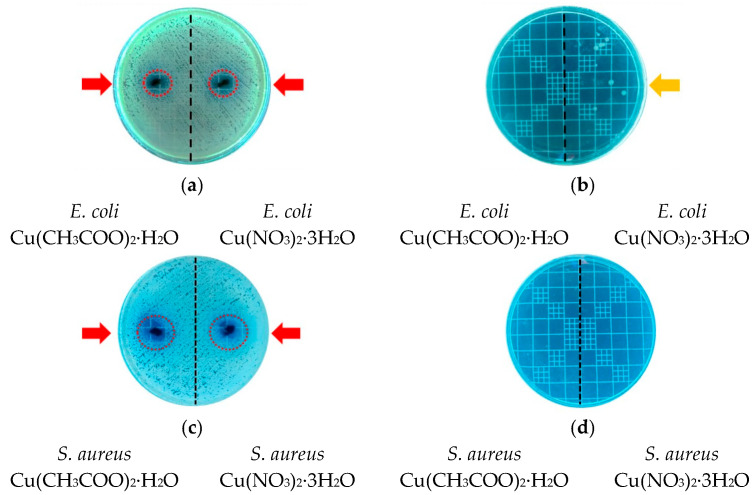
Antimicrobial activity of CuONPs synthesized at a pH of 7.0 using Cu(CH_3_COO)_2_·H_2_O and Cu(NO_3_)_2_·3H_2_O as copper precursors (10.0 g L^−1^) and calcined at 300 °C. Panels (**a**,**b**) show inhibition zone formation and liquid medium assays, respectively, against *E. coli*, while panels (**c**,**d**) show the corresponding results against *S. aureus*.

After the above experimental parameters were selected, the most suitable copper precursor salt for synthesis was chosen. The basis for selection was the antimicrobial performance of the samples obtained from each evaluated precursor salt. Thus, the antimicrobial activity of CuONPs synthesized at a pH of 7.0 and calcined at 300 °C was evaluated, and the results are presented in Figure 2. The formation of clear inhibition halos for the NPs synthesized using both copper precursors can be observed in Figure 2a,c (highlighted by dotted red circles and indicated by red arrows), demonstrating effective antimicrobial activity against *E. coli* and *S. aureus*. Moreover, these assays revealed that no bacterial colonies were observed after 24 h of contact with CuONPs synthesized from Cu(CH_3_COO)_2_·H_2_O, for either bacterial strain (Figure 2b,d). In contrast, the CuONPs obtained from Cu(NO_3_)_2_·3H_2_O provided a notable reduction in *E. coli* growth (as indicated by the yellow arrow) and complete inhibition of *S. aureus*. These results demonstrate that although both materials exhibit antimicrobial activity, the CuONPs synthesized from Cu(CH_3_COO)_2_·H_2_O exhibit superior performance, particularly against the Gram-negative strain.

Two CuONP samples were synthesized using a copper precursor concentration of 10.0 g L^−1^, which was identified as the minimum concentration needed to achieve detectable antimicrobial activity, on the basis of preliminary assays performed at lower precursor concentrations (Figure S3, Supplementary Material). In addition to influencing antimicrobial performance, the concentration of the copper precursor affects the NP size, which in turn plays a critical role in determining antimicrobial effectiveness. Several studies have revealed changes in the particle size as a function of the copper precursor concentration. Cerník and Thekkae Padil [52], for example, investigated the influence of the CuCl_2_ concentration on the synthesis of CuONPs and reported that precursor concentrations of 1.0, 2.0, and 3.0 mmol L^−1^ yielded NPs with average sizes of 7.8, 5.5, and 4.8 nm, respectively. Notably, the inhibition halos of the smallest particles were smaller than those of the larger particles (7.8 nm), indicating that antimicrobial activity does not necessarily increase with decreasing particle size. A similar trend may explain the behavior observed in this study.

At identical NP dosages (10.0 mg), the CuONPs synthesized using Cu(CH_3_COO)_2_·H_2_O completely inhibited the growth of the Gram-negative strain, whereas at least ten bacterial colonies were observed in media containing CuONPs synthesized from Cu(NO_3_)_2_·3H_2_O. These results indicate that Cu(CH_3_COO)_2_·H_2_O is a more suitable precursor for the green synthesis of CuONPs using *C. sinensis* leaf extract. This copper salt favors the formation of purer CuO-dominant NPs with enhanced antimicrobial performance, which is a key requirement for achieving the primary objective of this study. Therefore, subsequent studies on the synthesis mechanism, characterization, and incorporation into a chitosan polymeric matrix were conducted exclusively using samples obtained from Cu(CH_3_COO)_2_·H_2_O.

### 3.3. Proposed Formation Mechanism of CuONPs

**Figure 3:**
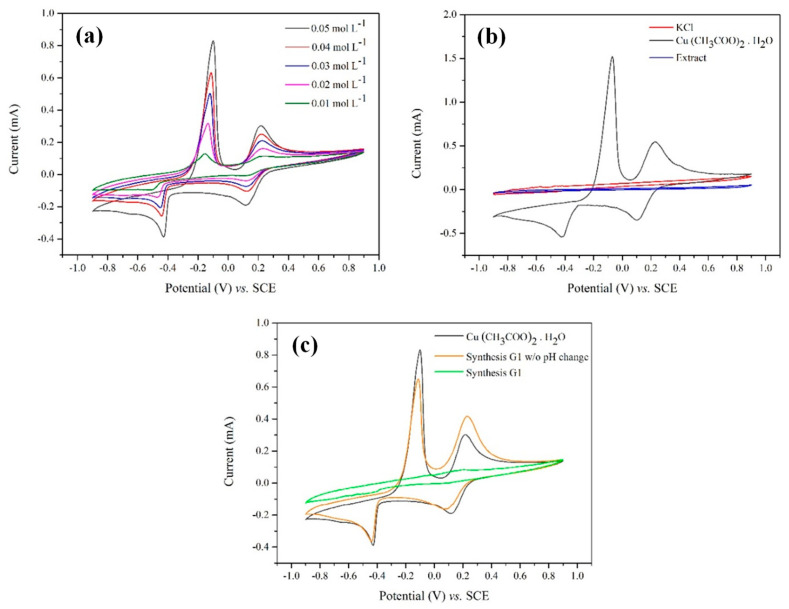
Cyclic voltammograms of (**a**) Cu(CH_3_COO)_2_·H_2_O at different concentrations; (**b**) supporting electrolyte (KCl) and *Citrus sinensis* leaf extract compared with Cu(CH_3_COO)_2_·H_2_O at the initial synthesis concentration; (**c**) sample obtained from green synthesis before heat treatment and from green synthesis without adjustment of the initial pH of the medium, both compared with Cu(CH_3_COO)_2_·H_2_O at the initial synthesis concentration.

To elucidate the formation mechanism of CuONPs prior to calcination, cyclic voltammetry was employed. This technique facilitates the investigation of oxidation-reduction processes associated with electron transfer reactions [53]. Measurements were performed by scanning the potential from the reduction region to the oxidation region, and the resulting cyclic voltammograms are shown in Figure 3.

The cyclic voltammograms of Cu(CH_3_COO)_2_·H_2_O at concentrations ranging from 0.05 to 0.01 mol L^−1^ are shown in Figure 3a. Two reduction and two oxidation peaks are observed, which can be assigned to the Cu(II)/Cu(I) and Cu(I)/Cu(0) redox transitions, respectively, which agrees with the behavior reported by Al-Harazie et al. [54]. These peaks correspond to the interconversion between the Cu(II)–Cu(I) and Cu(I)–Cu(0) species and provide a reference electrochemical signature for copper in solution.

To ensure that the observed electrochemical responses are solely associated with the copper species, the cyclic voltammograms of the supporting electrolyte (1.0 mol L^−1^ KCl) and *C. sinensis* leaf extract are plotted alongside the curve corresponding to the highest copper precursor concentration in Figure 3b. The absence of redox peaks confirms that neither the electrolyte nor the extract interferes with the electrochemical response of the copper species within the investigated potential window.

On the basis of these reference measurements, three cyclic voltammograms, all of which were obtained prior to calcination (one from the initial Cu(CH_3_COO)_2_·H_2_O solution, one from an aliquot collected after 4 h of reaction with the pH adjusted to 7.0, and another from an aliquot collected after 4 h of reaction without pH adjustment), are compared in Figure 3c. After interaction with the plant extract, a notable decrease in the redox peak intensity was observed in both cases. However, a clear difference emerged depending on the pH. When the pH of the synthesis medium was adjusted to 7.0, the redox peaks nearly disappeared after 4 h, indicating extensive transformation or consumption of electroactive copper species. In contrast, when the reaction was conducted at the natural pH of the solution, Cu(II)-related redox signals remained detectable even after 4 h of interaction.

These results demonstrate that the pH plays a key role in governing the transformation pathways of copper species during synthesis. The phenolic compounds present in the *C. sinensis* leaf extract, confirmed via Folin–Ciocalteu and DPPH analyses, are widely reported as effective chelating agents that can form coordination complexes with copper ions prior to NP formation. Under near-neutral conditions, changes in copper speciation favor the coexistence of CuOH^+^ species and the initial formation of partially suspended Cu(OH)_2_ [55], thereby enhancing the interactions between copper ions and plant metabolites.

According to Selvaraj et al. [56], flavonoids, one of the predominant classes of plant secondary metabolites, function as metal chelators through deprotonated phenolic OH groups and carbonyl functionalities. Within this context, the persistence of Cu(II) redox signals under natural pH conditions, in contrast to their disappearance at a pH of 7.0, indicates that pH adjustment promotes Cu(II) complexation and transformation rather than immediate reduction.

**Figure 4:**
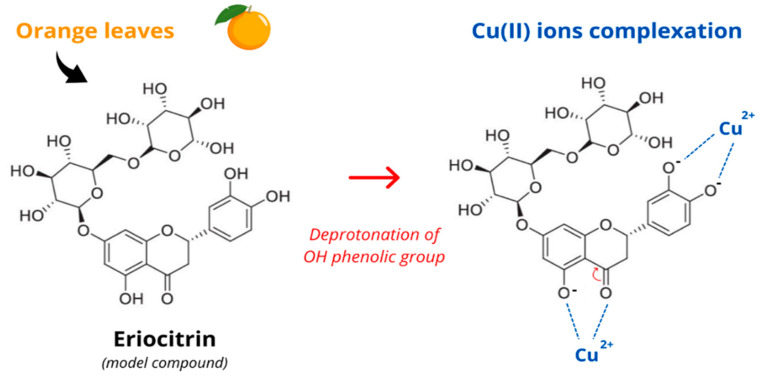
Proposed formation mechanism for CuONPs using eriocitrin as a model compound.

Considering these findings and the electrochemical results, a formation mechanism for CuONPs mediated by *C. sinensis* extract is proposed. As illustrated in Figure 4, the deprotonation of phenolic OH groups enhances their ability to coordinate with copper ions, facilitating reduction and complex stabilization. In this context, eriocitrin was selected as a representative model compound due to its high abundance in *C. sinensis* leaves [57], its well-defined polyphenolic structure containing multiple hydroxyl groups capable of metal chelation, its good solubility in aqueous and alcoholic media, and its reported thermal and chemical stability under extreme conditions [58].

### 3.4. Characterization of CuONPs

#### 3.4.1. Field Emission Scanning Electron Microscopy (FESEM) Coupled with Energy Dispersive Spectroscopy (EDS)

**Figure 5:**
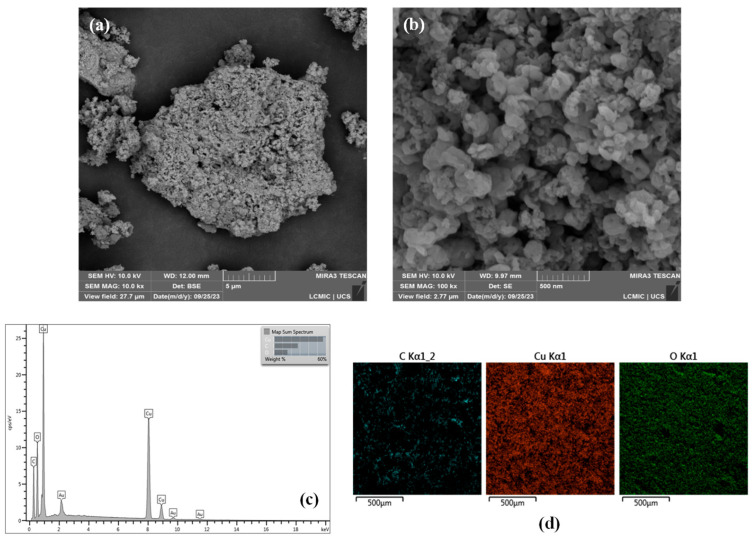
(**a**,**b**) FESEM micrographs, (**c**) EDS spectrum, and (**d**) elemental mapping of CuONPs synthesized at a pH of 7.0 using Cu(CH_3_COO)_2_·H_2_O as the copper precursor at a concentration of 10.0 g L^−1^ and calcined at 300 °C.

FESEM micrographs of CuONPs synthesized at a pH of 7.0 using Cu(CH_3_COO)_2_·H_2_O as the copper precursor at a concentration of 10.0 g L^−1^ and calcined at 300 °C are shown in Figure 5a,b.

As can be observed, the CuONPs exhibited a heterogeneous particle distribution, with notable variations in both the particle shape and size. This morphological heterogeneity is likely associated with the final stages of the synthesis process, including nucleation, growth, and thermal treatment. These morphological features are consistent with the findings of Sreeju et al. [59], who reported similar particle agglomeration and surface characteristics for CuONPs synthesized from *Psidium guajava* leaf extract.

The elemental composition of the synthesized CuONPs was analyzed via EDS. As shown in Figure 5c, the NPs comprised mainly Cu and O, confirming the formation of copper oxide. The presence of C and Au can be attributed to the sample preparation and analysis procedures, specifically the carbonaceous support and the gold sputter coating applied prior to FESEM analysis. Elemental mapping (Figure 5d) further confirmed the homogeneous spatial distributions of Cu and O throughout the analyzed region, indicating a uniform elemental composition of the synthesized CuONPs.

#### 3.4.2. Transmission Electron Microscopy (TEM)

**Figure 6:**
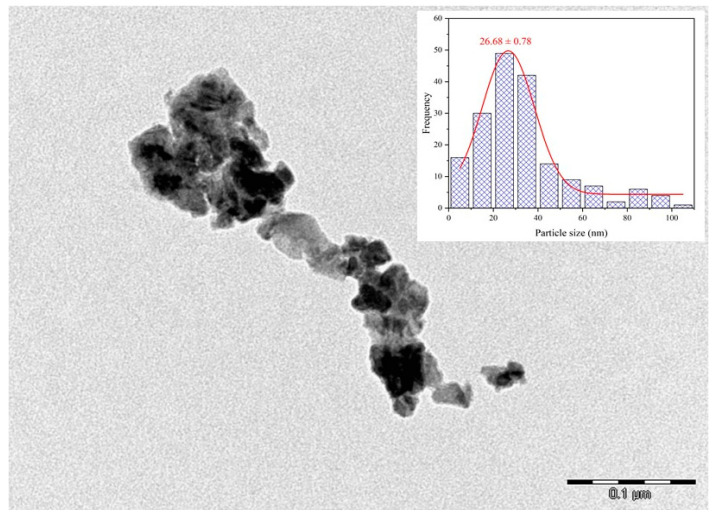
TEM image and particle size distribution histogram of CuONPs synthesized at a pH of 7.0 using Cu(CH_3_COO)_2_·H_2_O as the copper precursor at a concentration of 10.0 g L^−1^ and calcined at 300 °C.

To investigate the morphology and size of the CuONPs, TEM analysis was performed to complement FESEM analysis. A representative TEM image of the CuONPs synthesized at a pH of 7.0 using Cu(CH_3_COO)_2_·H_2_O as the copper precursor at a concentration of 10.0 g L^−1^ and calcined at 300 °C is shown in Figure 6. Consistent with the FESEM results, the NPs exhibited pronounced aggregation an did not display well-defined shapes.

The observed agglomeration can be attributed to the high surface energy of NPs and the relatively weak stabilization typically provided by phytochemicals present in plant extracts during green synthesis. In plant-mediated synthesis routes, compounds such as phenolics, flavonoids, and other secondary metabolites may act as natural capping agents [60]; however, their stabilizing effect is generally weaker than that of synthetic surfactants or polymeric stabilizers commonly used in conventional NP synthesis [47,48]. As a result, a certain degree of particle aggregation is frequently observed. In addition, synthesis parameters such as precursor concentration, extract composition, pH, reaction temperature, and calcination conditions can influence nucleation and growth processes and therefore affect the final degree of NP agglomeration [60].

TEM analysis also facilitated the estimation of the particle size distribution (inset of Figure 6). The resulting histogram revealed a broad size range, spanning from 1 to 110 nm, with the majority of particles distributed between 20 and 30 nm. This size range is particularly relevant for antimicrobial applications [61]. As commonly reported, CuONPs obtained via green synthesis routes typically exhibit high agglomeration, which can impede accurate size determination. Consequently, the measurement uncertainties may be greater, potentially leading to an underestimation of the actual particle size distribution [62]. Comparable morphological features and particle sizes have been reported by Sreeju et al. [59], who synthesized CuONPs using *P. guajava* leaf extract. In that study, the NPs exhibited an average diameter of approximately 37 nm, a largely spherical morphology, and notable aggregation, which suitably agree with the observations reported here.

### 3.5. Chitosan–CuONP Composites

To evaluate the performance of the CuONPs synthesized at a pH of 7.0 using Cu(CH_3_COO)_2_·H_2_O as the copper precursor at a concentration of 10.0 g L^−1^ and calcined at 300 °C, chitosan–CuONP composites were prepared and tested against *E. coli* and *S. aureus* via the disc diffusion method. Chitosan was selected as the support material because of its intrinsic antimicrobial and antifungal properties [63,64], which can act synergistically with the antimicrobial activity of CuONPs. This polymer is widely used in medical and food-related applications and has received increasing attention as a sustainable material for wastewater treatment systems [65].

**Figure 7:**
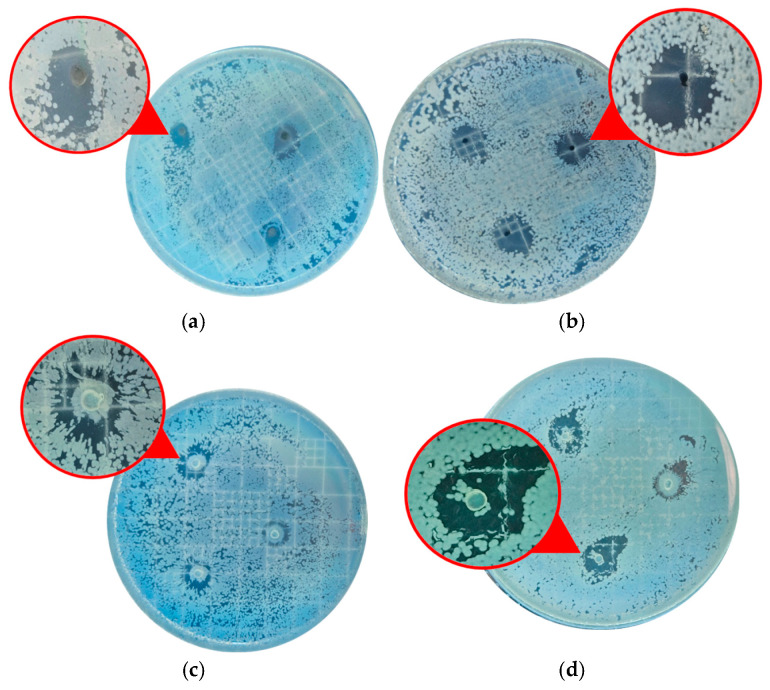
Evaluation of the antimicrobial activity of chitosan–CuONP composites (**a**,**b**) and pure chitosan (**c**,**d**) against *S. aureus* and *E. coli* strains.

The results obtained for the chitosan–CuONP composites and pure chitosan are shown in Figure 7. In general, well-defined inhibition halos were observed around the composite pellets for both bacterial strains, indicating notable antimicrobial activity. In contrast, pure chitosan exhibited limited performance, as bacterial colonies were still observed near the pellets. These results clearly demonstrate that the incorporation of CuONPs significantly enhances the antimicrobial properties of the chitosan polymeric matrix.

The antimicrobial activity of CuONPs has been attributed to several complementary mechanisms reported in the literature. One of the most frequently proposed mechanisms involves the release of copper ions from the NP surface, which can penetrate bacterial cells and interact with proteins, enzymes, and nucleic acids, disrupting essential metabolic processes [66]. In addition, CuONPs may promote the generation of ROS, which induce oxidative stress and damage cellular components such as lipids, proteins, and DNA [66,67,68]. Another possible mechanism involves the direct interaction of NPs with the bacterial cell membrane, leading to structural damage, increased membrane permeability, and eventual cell death [66]. These mechanisms may occur simultaneously and contribute collectively to the antimicrobial activity of CuO nanomaterials.

These findings are consistent with those of previous reports. Eltz et al. [69] incorporated AgNPs and CuONPs into polymeric matrices based on poly(allylamine hydrochloride) and poly(acrylic acid) for industrial wastewater treatment applications and demonstrated effective microbial inhibition even at low metal loadings after 30 and 120 min of contact. Tran et al. [70] reported that CuONPs incorporated into cellulose- and chitosan-based composites exhibited significant antimicrobial activity against both Gram-positive (vancomycin-resistant *Enterococcus* and *Streptococcus agalactiae*) and Gram-negative (*E. coli* and *Enterobacter cloacae*) bacteria. Similarly, Farhoudian et al. [71] reported comparable antimicrobial performance levels for chitosan–CuO nanocomposites tested against *E. coli* and *S. aureus*. In that study, the antimicrobial efficiency of the nanocomposite hydrogels depended primarily on the concentration of the incorporated CuONPs, irrespective of the bacterial strain.

Moreover, chitosan-immobilized CuONPs generally exhibit higher antimicrobial activity against Gram-positive bacteria, such as *S. aureus*, than against Gram-negative strains. This behavior has been attributed to the higher density of reactive functional groups (e.g., amine and carboxyl groups) in Gram-positive cell walls, which enhances the interactions with copper species. In contrast, the outer membrane characteristic of Gram-negative bacteria can restrict the penetration and action of antimicrobial agents [72,73].

## 4. Conclusions

In this study, the viability of *C. sinensis* leaf extract as an efficient and sustainable reducing agent for green synthesis of CuONPs was demonstrated. The extract exhibited a high phenolic content (400.90 ± 3.60 µg GAE mL^−1^) and high antioxidant capacity (≈80% DPPH inhibition), confirming its suitability for mediating NP formation. The optimized extraction conditions involved leaf drying and grinding, the use of water as a green solvent, and extraction at 70 °C for 30 min.

The optimization of the synthesis parameters revealed that the pH, copper precursor concentration, and calcination temperature greatly influence the phase composition, structure, and antimicrobial performance of the resulting CuONPs. Under the optimal conditions (pH 7.0, calcination temperature of 300 °C, and 10.0 g L^−1^ of Cu(CH_3_COO)_2_·H_2_O), CuO-dominant NPs with high antimicrobial activity were obtained. Cyclic voltammetry provided additional insight into the formation mechanism, highlighting the interaction between copper ions and extract metabolites and the key role of pH modulation during synthesis. The CuONPs, predominantly within the 20–30 nm size range, were successfully immobilized in a chitosan polymeric matrix, resulting in composites that preserved the antimicrobial activity of the NPs and significantly enhanced the antibacterial performance of the biopolymer, thus demonstrating the synergistic effect between chitosan and CuONPs.

Although the composites were not evaluated in real application systems, such as wastewater disinfection, the results demonstrated that the combination of green-synthesized CuONPs with chitosan yields materials with relevant antimicrobial properties. Despite this promising performance, additional studies are required to evaluate their stability, reusability, copper release behavior, and antimicrobial activity in complex matrices representative of industrial wastewater. Overall, this work establishes an environmentally friendly methodology for producing CuONPs and incorporating them into chitosan-based materials, providing a solid foundation for future investigations targeting advanced water disinfection technologies and other environmentally relevant applications.

## Figures and Tables

**Table 1:** Evaluated extraction conditions and experimental parameters for each extract analyzed.

Test	Sample	Experimental Parameters			
		Leaf Preparation	Solvent	Temperature (°C)	Time (min)
1	A1	Fresh	Ethanol/deionized water (1:1 *v*/*v*)	25	30
	B1	Dried	Ethanol/deionized water (1:1 *v*/*v*)	25	30
	C1	Fresh	Deionized water	70	30
	D1	Dried	Deionized water	70	30
2	A2	Dried	Deionized water	25	30
	B2	Dried	Deionized water	40	30
	C2	Dried	Deionized water	70	30
3	A3	Dried	Deionized water	70	5
	B3	Dried	Deionized water	70	10
	C3	Dried	Deionized water	70	30

**Table 2:** Methodologies evaluated for the green synthesis of CuONPs using *Citrus sinensis* extract.

Synthesis *	Experimental Parameters			
	Precursor Salt	Precursor Salt Concentration (g L^−1^)	pH	Calcination Temperature (°C)
A1 **	Cu(CH_3_COO)_2_·H_2_O	5.0	3.0	-
A2 **	Cu(NO_3_)_2_·3H_2_O	5.0	3.0	-
B1	Cu(CH_3_COO)_2_·H_2_O	5.0	7.0	-
B2	Cu(NO_3_)_2_·3H_2_O	5.0	7.0	-
C1	Cu(CH_3_COO)_2_·H_2_O	5.0	10.0	-
C2	Cu(NO_3_)_2_·3H_2_O	5.0	10.0	-
D1	Cu(CH_3_COO)_2_·H_2_O	5.0	7.0	400
D2	Cu(NO_3_)_2_·3H_2_O	5.0	7.0	400
E1	Cu(CH_3_COO)_2_·H_2_O	5.0	10.0	400
E2	Cu(NO_3_)_2_·3H_2_O	5.0	10.0	400
F1	Cu(CH_3_COO)_2_·H_2_O	5.0	7.0	200
F2	Cu(NO_3_)_2_·3H_2_O	5.0	7.0	200
G1	Cu(CH_3_COO)_2_·H_2_O	10.0	7.0	300
G2	Cu(NO_3_)_2_·3H_2_O	10.0	7.0	300

* All syntheses were performed using *Citrus sinensis* leaf extract at a concentration of 0.1 g mL^−1^. ** Syntheses “A1” and “A2” did not yield sufficient CuONPs for analysis.

**Table 3:** Influences of experimental parameters on extract preparation: Test 1, drying of *Citrus sinensis* leaves and solvent type; Test 2, solvent temperature; and Test 3, contact time.

Test	Sample	Leaf Preparation	Solvent	Temperature (°C)	Time (min)	GAE (µg mL^−1^)
1	A1	Fresh	Ethanol/deionized water (1:1 *v*/*v*)	25	30	72.61 ± 1.24
	B1	Dried	Ethanol/deionized water (1:1 *v*/*v*)	25	30	386.49 ± 0.801
	C1	Fresh	Deionized water	70	30	365.77 ± 8.61
	D1	Dried	Deionized water	70	30	400.90 ± 3.60
2	A2	Dried	Deionized water	25	30	358.56 ± 2.58
	B2	Dried	Deionized water	40	30	366.67 ± 2.60
	C2	Dried	Deionized water	70	30	400.90 ± 3.60
3	A3	Dried	Deionized water	70	5	325.77 ± 0.910
	B3	Dried	Deionized water	70	10	323.96 ± 0.721
	C3	Dried	Deionized water	70	30	400.90 ± 3.60

## Data Availability

Data will be made available upon request.

## Supplementary Materials

The following supporting information can be downloaded at https://www.mdpi.com/article/10.3390/nano16060369/s1. Figure S1: X-ray diffraction diffractograms of CuONPs obtained from the “B”, “C”, ‘D”, “E”, and “F” syntheses: (a) B1; (b) D1; (c) B2; (d) D2; (e) C1; (f) E1; (g) C2; and (h) E2; Figure S2: XRD diffractograms of CuONPs obtained from the “F” synthesis at pH 7.0, followed by calcination at 200 °C: (a) F1; (b) F2; (c) reference diffraction patterns of CuO; and (d) reference diffraction patterns of Cu_2_O; Figure S3: Antimicrobial tests with CuONPs obtained from the “D”, “E”, and “F” synthesis. Panels (a), (b), (e), (f), (i), and (j) show the results for antimicrobial inhibition and bactericidal activity against the *E. coli* strain, respectively; while panels (c), (d), (g), (h), (k), and (l) present the same results against the *S. aureus* strain, respectively.

## Abbreviations

The following abbreviations are used in this manuscript:

CFU	Colony-forming units
CLSI	Clinical and Laboratory Standards Institute
*C. sinensis*	*Citrus sinensis*
CuONPs	Cupric oxide nanoparticles
DNA	Deoxyribonucleic acid
DPPH	2,2-diphenyl-1-picrylhydrazyl
*E. coli*	*Escherichia coli*
EDS	Coupled with energy dispersive spectroscopy
FESEM	Field emission scanning electron microscopy
GA	Gallic acid
GAE	Gallic acid equivalent
NPs	Nanoparticles
ROS	Reactive oxygen species
*S. aureus*	*Staphylococcus aureus*
TEM	Transmission electron microscopy
XRD	X-ray diffraction
