# Supplementary material for: Green Synthesis of Copper Oxide Nanoparticles Using Citrus sinensis Leaves: Effects of Experimental Parameters, Antimicrobial Evaluation and Development of Chitosan Composites
Source: Nanomaterials (Basel). 2026 Mar 18;16(6):369. doi: 10.3390/nano16060369 (PMC13029666; doi:10.3390/nano16060369)
Supplement: Supplementary file 1 [file nanomaterials-16-00369-s001.zip › nanomaterials-4191858-supplementary.pdf]

nanomaterials

Supplementary Material

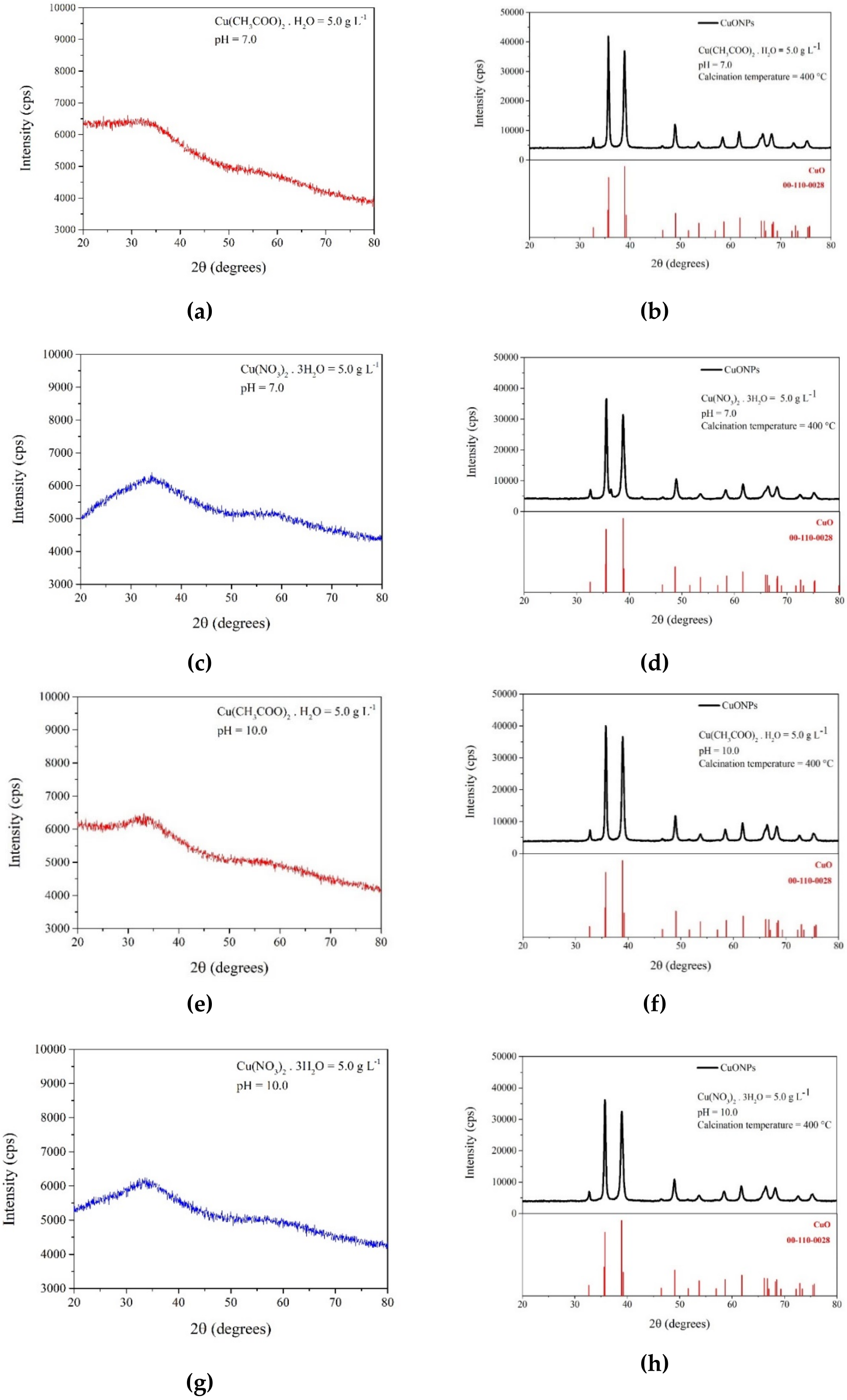

Figure S1. X-ray diffraction diffractograms of CuONPs obtained from the "B", "C", 'D", "E", and "F" syntheses: (**a**) B1; (**b**) D1; (**c**) B2; (**d**) D2; (**e**) C1; (**f**) E1; (**g**) C2; and (**h**) E2

(a)

(b)

(c)

(d)

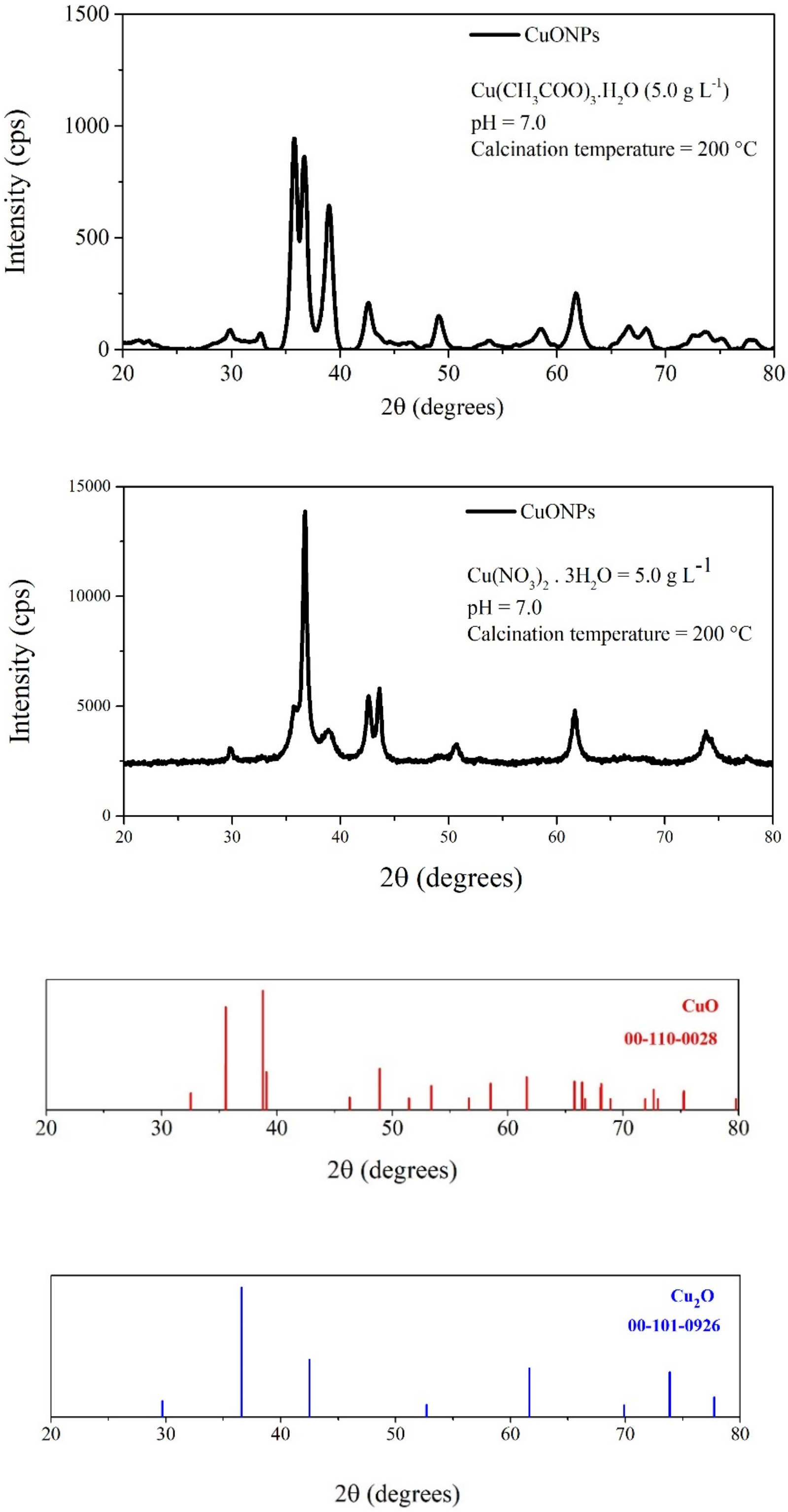

Figure S2. XRD diffractograms of CuONPs obtained from the "F" synthesis at pH 7.0, followed by calcination at 200 °C: (**a**) F1; (**b**) F2; (**c**) reference diffraction patterns of CuO; and (**d**) reference diffraction patterns of Cu_2O

https://doi.org/10.3390/nano16060369

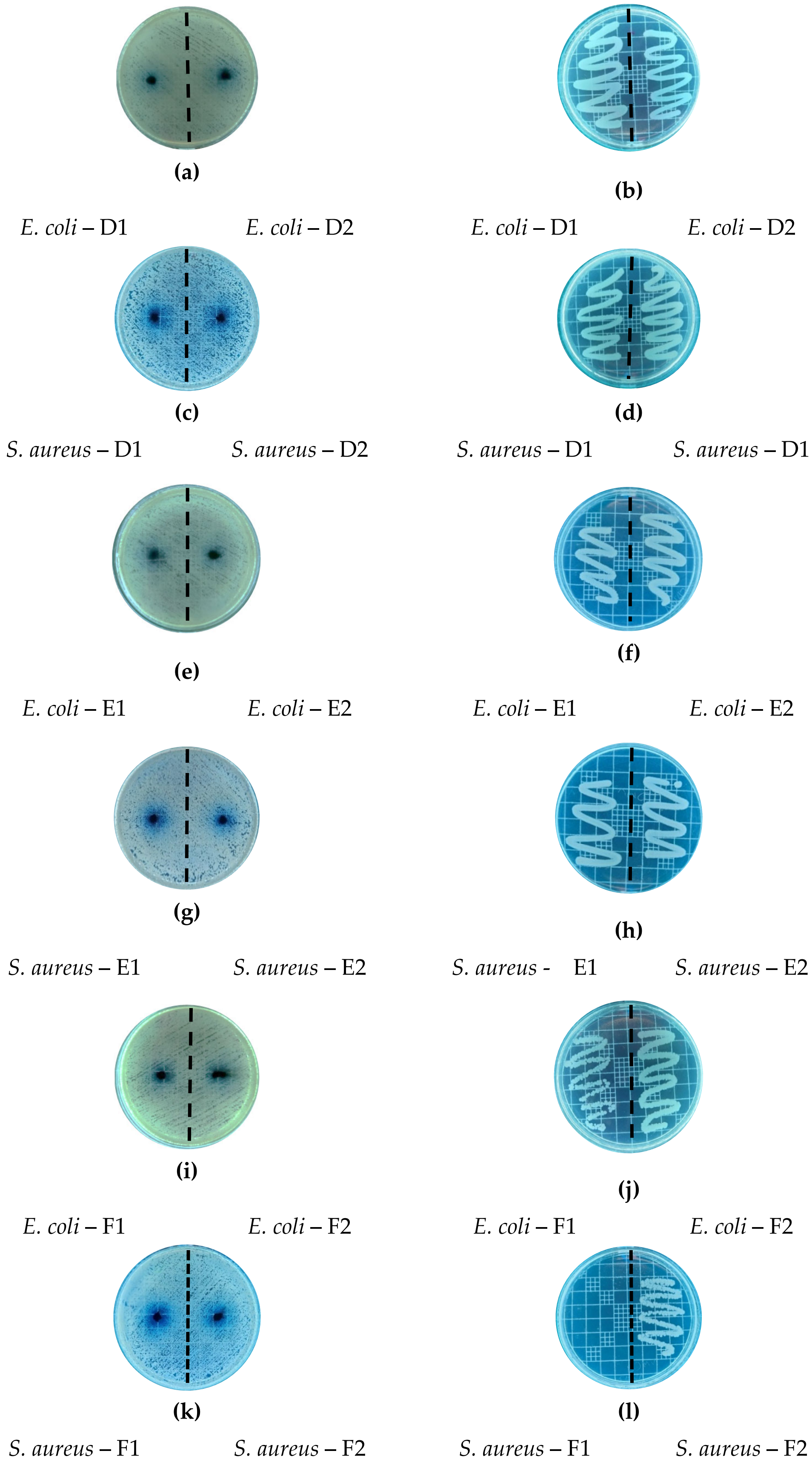

Figure S3. Antimicrobial tests with CuONPs obtained from the "D", "E", and "F" synthesis. Panels (**a**), (**b**), (**e**), (**f**), (**i**), and (**j**) show the results for antimicrobial inhibition and bactericidal activity against the *E. coli* strain, respectively; while panels (**c**), (**d**), (**g**), (**h**), (**k**), and (**l**) present the same results against the *S. aureus* strain, respectively

https://doi.org/10.3390/nano16060369

Disclaimer/Publisher's Note: The statements, opinions and data contained in all publications are solely those of the individual author(s) and contributor(s) and not of MDPI and/or the editor(s). MDPI and/or the editor(s) disclaim responsibility for any injury to people or property resulting from any ideas, methods, instructions or products referred to in the content.

https://doi.org/10.3390/nano16060369
